# Marked reduction of cerebellar deficits in upper limbs following transcranial cerebello-cerebral DC stimulation: tremor reduction and re-programming of the timing of antagonist commands

**DOI:** 10.3389/fnsys.2014.00009

**Published:** 2014-01-30

**Authors:** Giuliana Grimaldi, Nordeyn Oulad Ben Taib, Mario Manto, Florian Bodranghien

**Affiliations:** ^1^Unité d'Etude du Mouvement, ULB NeurologieBruxelles, Belgium; ^2^Service de Neurochirurgie, CHU Saint-PierreBruxelles, Belgium; ^3^Fonds de la Recherche Scientifique-ULBBruxelles, Belgium

**Keywords:** DC stimulation, cerebellum, ataxia, tremor, hypermetria, antagonist, EMG, timing

## Abstract

Cerebellar ataxias represent a very heterogeneous group of disabling disorders for which we lack effective symptomatic therapies in most cases. There is currently an intense interest in the use of non-invasive transcranial DC stimulation (tDCS) to modulate the activity of the cerebellum in ataxic disorders. We performed a detailed laboratory assessment of the effects of transcranial cerebello-cerebral DC stimulation (tCCDCS, including a sham procedure) on upper limb tremor and dysmetria in 2 patients presenting a dominant spinocerebellar ataxia (SCA) type 2, one of the most common SCAs encountered during practice. Both patients had a very similar triplet expansion size in the ATXN2 gene (respectively, 39 and 40 triplets). tCCDCS reduced both postural tremor and action tremor, as confirmed by spectral analysis. Quadratical PSD (power spectral density) of postural tremor dropped to 38.63 and 41.42% of baseline values in patient 1 and 2, respectively. The integral of the subband 4–20 Hz dropped to 46.9 and 62.3% of baseline values, respectively. Remarkably, tCCDCS canceled hypermetria and reduced dramatically the onset latency of the antagonist EMG activity associated with fast goal-directed movements toward 3 aimed targets (0.2, 0.3, and 0.4 rad). Following tCCDCS, the latency dropped from 108–98 to 63–57 ms in patient 1, and from 74–87 to 41–46 ms in patient 2 (mean control values ± *SD*: 36 ± 8 to 45 ± 11 ms), corresponding to a major drop of z scores for the 2 patients from 7.12 ± 0.69 to 1.28 ± 1.27 (sham procedure: 6.79 ± 0.71). This is the first demonstration that tCCDCS improves upper limb tremor and hypermetria in SCA type 2. In particular, this is the first report of a favorable effect on the onset latency of the antagonist EMG activity, a neurophysiological marker of the defect in programming of *timing* of motor commands. Our results indicate that tCCDCS should be considered in the symptomatic management of upper limb motor deficits in cerebellar ataxias. Future studies addressing a tDCS-based neuromodulation to improve motor control of upper limbs are required (a) in a large group of cerebellar disorders, and (b) in different subgroups of ataxic patients. The anatomical location of the cerebellum below the skull is particularly well suited for such studies.

## Introduction

Cerebellar ataxias represent a very heterogeneous group of sporadic and genetic disabling diseases (Manto and Marmolino, 2009). Voluntary movement of the limbs is typically jerky (Holmes, 1917). Tremor and dysmetria are two main clinical manifestations which contribute to disability during daily life (Saute et al., 2012). Although the number of defined cerebellar ataxias is growing thanks to genetic discoveries, we still lack efficient symptomatic therapies for the majority of them, hence the current intense research effort to identify novel strategies aiming to reduce the motor deficits, to promote learning and to increase the effects of rehabilitation (Hamada et al., 2012; Grimaldi and Manto, 2013).

There is a growing interest in using transcranial DC stimulation (tDCS) to modulate cerebellar functions (Galea et al., 2009; Pope and Miall, 2012; Ferrucci and Priori, 2014). This non-invasive technique is based on the application of a steady current of small intensity (usually between 0.5 and 2 mAmp; anodal or cathodal) between 2 large electrodes fixed on the scalp (Tomlinson et al., 2013). The current, administered either in a continuous or in an intermittent mode, causes a polarity-dependent modulation of brain activity which is site-specific (Nitsche et al., 2003). tDCS of the cerebellum is particularly attractive also because of the anatomical location of the cerebellar cortex immediately below the skull. It has been shown that anodal tDCS of the cerebellum increases the excitability of the cerebellar cortex in human, thus reinforcing the inhibitory effect exerted by the cerebellar cortex over cerebellar nuclei (Galea et al., 2009). These latter project to the contralateral motor cortex mainly via the cerebello-thalamo-cortical pathway. Hypoexcitability of the motor cortex with enhanced intra-cortical inhibition is a major defect commonly observed in cerebellar ataxias and contributes to the deficits of motor skills (Wessel et al., 1996; Tamburin et al., 2004). Therefore, one therapeutic possibility to reduce cerebellar ataxia could be the neuromodulation of the excitability of the cerebello-thalamo-cortical pathway by acting on the cerebellar cortex and/or the contralateral motor cortex. In a recent report including a sham procedure, Pozzi et al. have shown that anodal tDCS of the primary motor cortex improves gait asymmetry in sporadic ataxia or in the dominant SCA type 6 (Pozzi et al., 2013). The combination of tDCS of the cerebellum with tDCS of the contralateral motor/premotor cortex appears as an interesting option (Grimaldi and Manto, 2013). Indeed, experimental studies in rodents show that (1) anodal tDCS of the motor cortex restores the excitability of the motor cortex in case of extensive damage of the contralateral cerebellar hemisphere (Oulad Ben Taib and Manto, 2009), (2) epidural DCS of the cerebellum reshapes the corticomotor maps of couples of agonist/antagonist muscles in limbs, enhances the spinocerebellar evoked potentials associated with peripheral electrical stimulation and augments cerebellar blood flow both at the level of cerebellar cortex and cerebellar nuclei likely by acting on the neurovascular coupling (Grimaldi and Manto, 2013; Oulad Ben Taib and Manto, 2013); Oulad Ben Taib and Manto, in preparation), (3) epidural DCS of the cerebellum followed by tDCS of the contralateral motor cortex is very efficient to modulate the corticomotor output which is normally associated with repetitive electrical stimulation of the sciatic nerve (Grimaldi and Manto, 2013; Oulad Ben Taib and Manto, 2013), and (4) the sequence epidural DCS of the cerebellum followed by epidural DCS of the contralateral motor cortex is more efficient than the reverse when attempting to restore the adaptation of the corticomotor responses to peripheral repetitive stimulation in the model of high frequency electrical stimulation of the interpositus nucleus (Oulad Ben Taib et al., 2005a,b; Oulad Ben Taib and Manto, in preparation). Based on findings in rodents and on the work of Pozzi et al., we tested the hypothesis that tDCS applied over the cerebellum immediately followed by tDCS applied over the contralateral motor cortex (tCCDCS: transcranial cerebello-cerebral DC stimulation) could antagonize upper limb tremor and dysmetria in patients with a commonly encountered SCA and who exhibited a predominant cerebellar syndrome.

## Methods

### Patients

**Figure 1:**
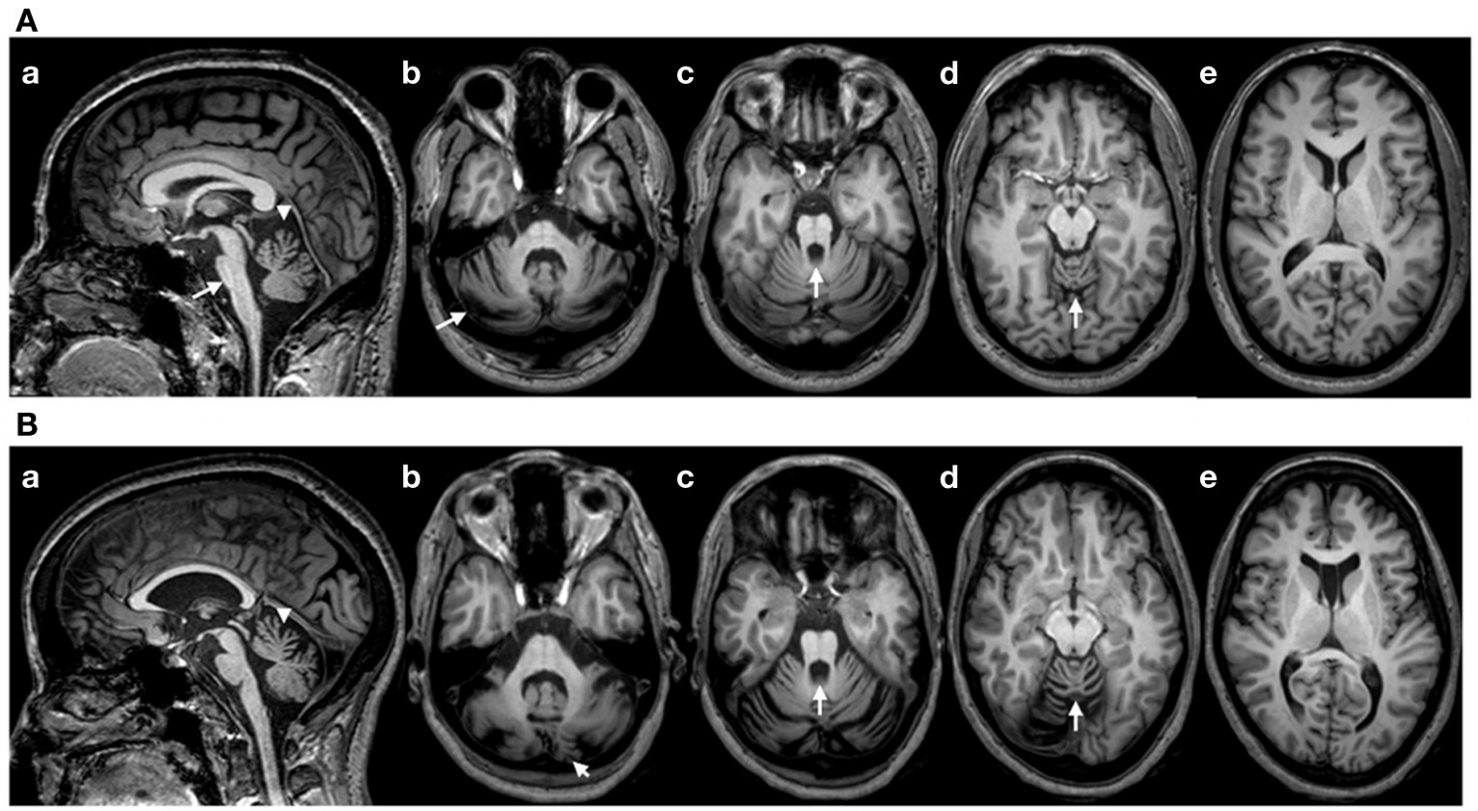
**(A)** Brain MRI (T1-weighted images) in patient 1. (a): sagittal image showing a flattening of the pons (arrow), atrophy of the upper vermis (arrowhead) and a thin spinal cord in upper cervical segments; (b–d): axial sections showing atrophy of the cerebellum at the level of the middle cerebellar peduncle (b; arrow: atrophy of the cerebellar cortex), the upper pons with enlargement of the 4th ventricle (c; arrow) and the upper vermis (d; arrow). (e): Normal appearance of the cerebral cortex (axial slice at the level of the head of caudate nucleus). **(B)** Brain MRI in patient 2 (T1-weighted images). Flattening of the pons is less severe as compared to patient 1 (a), whereas the degree of cerebellar atrophy is relatively similar (b–e). Note the presence of a cavum of the septum pellucidum in patient 2.

*Patient 1:* This right-handed 49-year-old right-handed woman had a 5 years history of gait difficulties, in a context of familial dominant ataxia. In addition to cerebellar signs (see ataxia rating score in the Results section), neurological examination showed impaired horizontal saccades (decreased velocities; very suggestive of the disorder) and decreased tendon reflexes in 4 limbs. Detailed sensory examination (position sense, vibration, tactile sensation) and evaluation of motor strength (5/5 on MRC scale) were normal in upper limbs. Hoffmann's reflex was negative. Brain MRI showed atrophy of the cerebellum and brainstem, extending to the cervical segments of the spinal cord (Figure 1A). We did not apply voxel-based morphometry (VBM) or 3D-based volumetry to quantify the atrophy. Genetic study for SCA type 2 (ATXN2 gene; chromosome 12q23-24.1) showed 22 and 39 CAG triplets (*N* < 32) (Gispert et al., 1993). *Patient 2*: this right-handed 43-year-old right-handed man had a 14 years history of loss of balance, in a context of familial dominant ataxia. In addition to cerebellar signs, neurological examination showed slowing of horizontal saccades and decreased tendon reflexes in 4 limbs. Detailed sensory examination and evaluation of motor strength were normal in upper limbs. Hoffmann's reflex was negative. Brain MRI showed atrophy of the cerebellum and brainstem (Figure 1B; no VBM or 3D-based volumetry performed). Genetic study for SCA type 2 showed 22 and 40 triplets.

We selected these patients because (a) they both presented deficits of motor control in upper limbs characterized by a postural/action tremor steady in amplitude and dysmetria (these are clinical features commonly observed in SCA2), (b) they both exhibited a delayed onset latency of the antagonist EMG activity associated with fast goal-directed movements (this delay is insensitive to practice trials), and (c) the size of the triplet expansion was very similar.

The study was approved by the local ethical committee of ULB and both patients signed a written informed consent following full explanation of the experimental procedures.

### tCCDCS

Data were collected on right upper limb in 3 experimental conditions: (1) at baseline, (2) after a sham stimulation (20 min of sham stimulation over the cerebellum, immediately followed by sham stimulation over the contralateral motor cortex—see below), and (3) after tCCDCS (20 min of anodal stimulation over the cerebellum, immediately followed by anodal stimulation over the contralateral motor cortex). The rationale for this design baseline-sham-tDCS in cerebellar disorders has been explained elsewhere (Grimaldi and Manto, 2013). We did not observe any effect on upper limb movements by the sole anodal tDCS of the cerebellum in a previous study. We applied an “off-line” methodology for the recordings of tremor and fast goal-directed movements (assessment within 45 min after application of sham or tCCDCS; we attempted to avoid the occurrence of fatigue which occurs typically when recordings sessions last more than 3 h in cerebellar patients). This off-line approach is commonly applied in tDCS studies.

For sham and anodal stimulation of the cerebellum, the anode—a sponge electrode; size: 50 × 40 mm—was positioned at the level of the posterior fossa on the right side with the center of the sponge at about 3 cm to the right of the inion, in order to target the right cerebellar hemisphere, given the lateralized cerebellar functions for upper limbs (Galea et al., 2009). The second sponge electrode—the cathode; same size than anode—was applied over the contralateral supra-orbital area. Electrodes were soaked (with a solution of NaCl 0.9%). The period of stimulation lasted 20 min both for sham stimulation and anodal stimulation. Subsequently, the anode was positioned over the hand representation of left primary motor cortex, with the cathode placed over the right supra-orbital area (this selection is based on our experience in rodents). A second period of stimulation of 20 min for both conditions (sham and active stimulation) was used. Current delivered was 1 mAmp (portable stimulator with a 9 V battery; CES, Canada). Current was increased gradually from 0 to 1 mAmp over 30 s, as confirmed by the analysis of the current using a Fluke PM3384A Comb scope. For sham stimulation, once the current reached the plateau, it was gradually decreased to zero over a period of about 1 min, so that patients were blinded as to whether they were receiving sham stimulation or anodal tDCS.

Upper limb tremor (postural and action) and dysmetria were studied at baseline, post-sham and post-tCCDCS. We did not assess kinetic tremor because it was more variable in our patients as compared to postural and action tremor which were both steady.

### Clinical evaluation

An ataxia rating scale (SARA; scale designed for dominant ataxias—higher values correspond to a lower performance) was applied at baseline, immediately after sham and immediately after tCCDCS.

### Upper limb tremor

#### Postural tremor

Patients were comfortably seated in an armchair, and equipped with 2 triaxial accelerometers, located at the right index finger and at the right metacarpal region (see Grimaldi et al., 2013). They were asked to maintain the upper limbs motionless horizontally in front of them and parallel to the floor. Three recordings of 15 s were performed at baseline, after sham and post-tCCDCS. The sampling rate was 512 Hz per axis for each accelerometer. For signal processing of accelerometry signals, we computed the Root Mean Square (RMS) and the total traveled distance (expressed in mm). We performed the spectral analysis using Fast Fourier Transform (FFT) as recommended, using Matlab (MathWorks, USA) (Grimaldi and Manto, 2010; McNames, 2013). The 15 s time-series were segmented in 5 segments. Auto-spectra of 5 sequential 3 s data epochs were averaged to produce smoothed autospectra, with mean removal and a windowing (Hamming) for each data segment (Grimaldi and Manto, 2010). The following spectral parameters were extracted and means were computed for the 3 experimental conditions (baseline, post-sham and post-tCCDCS): maximal PSD (maxPSD), peak frequency of power spectra (PFr), crest factor in the 4–20 Hz frequency band (CF = the ratio of maximal PSD divided by the integral of the 4–20 Hz frequency sub-band), center frequency (median value of the area below the power spectrum) (Grimaldi and Manto, 2008; Grimaldi et al., 2013). Composite data (square root of the sum of the accelerations squared for all three axis) were processed as reported earlier (Grimaldi et al., 2013; Shaikh et al., 2008). Data presented in the results section are mean ± standard deviation (*SD*) of the 3 recordings performed in each experimental condition.

#### Action tremor

Patients were asked to maintain the manipulandum of a mechatronic myohaptic device specifically designed and built for wrist motion analysis (see also next section). Patients were asked to keep the manipulandum motionless during 16 s. The manipulandum exerted a constant extensor torque of 0.5 Nm from the neutral position, thus eliciting an action tremor. Patients were allowed to perform 5 practice trials before recordings. This number of practice trials is based on our experience with ataxic patients (patients are familiar with the test after 2–3 trials). Sampling rate for position signal was 2048 Hz. We extracted the short-time Fourier transform (STFT) using Igor Pro 6.01 software (Wavemetrics, USA). The 16 s time-series were filtered (IIR high-pass 2 Hz) and segmented in 8 segments. Auto-spectra of 8 sequential data epochs of 2 s were averaged. We computed maxPSD, PFr, CF in the 4–20 Hz frequency sub-band, integrals of frequency sub-bands (2–4, 4–8, 8–12, 12–16, 4–20 Hz). Because cerebellar action tremor is often more severe at the frequency of 3 Hz, we also extracted the tremor data in the sub-band 2.5–3.5 Hz by applying a corresponding band-pass IIR filter of order 4. The RMS was also computed on the filtered data.

#### Relative entropy of postural and action tremor

Cerebellum is well known to contribute to timing of motor commands and is considered as a “clock” in the brain (Braitenberg, 1967; Ivry and Spencer, 2004). Therefore, in order to measure the possible effectiveness of tCCDCS on the randomness of spectral data, we computed the Kullback-Leibler entropy, a method used to measure the degree of similarity between 2 spectrograms (Freeman and Quian Quiroga, 2013). The relative entropy was computed using the basal state as the reference (post-sham condition vs. basal condition, and post-tCCDCS vs. basal condition). The following formula was used (Freeman and Quian Quiroga, 2013):

$$K(p|q)=\sum_{k}p_{k}\log_{2}\frac{p_{k}}{q_{k}}$$

where:

*p*_*k*_ is the frequency probability of the post-sham (or post-tCCDCS) signal.

*q*_*k*_ is the frequency probability of baseline signal.

### Dysmetria

We investigated fast goal-directed pointing single-joint movements with the haptic technology as reported earlier (Manto et al., 2010). The range of motion was constrained mechanically from −1 rad to +1 rad. Movements were studied in the free mode of the manipulandum at a sampling rate of 2048 Hz. The 2 patients performed sets of 10 fast pointing movements over 3 distances (targets displayed on a computer screen in front of the patient; 3 targets: 0.2, 0.3, 0.4 rad) following 3–4 practice trials. Subjects were comfortably seated, with the shoulder relaxed and the upper arm perpendicular to the forearm. The hand and forearm were affixed with straps. The wrist joint was carefully aligned with the motor axis. Movements were performed in the horizontal plane. We recorded the surface EMG activities of the flexor carpi radialis (FCR; agonist) and extensor carpi radialis (ECR; antagonist) muscles. Surface EMG activities were amplified (× 1000) and full-wave rectified (filter settings: 20–500 Hz; Delsys surface electrodes, USA; electrodes fixed on the skin with tape). We averaged each set of 10 movements, both for wrist angle data and EMG data. Individual records were aligned to the onset of the agonist EMG burst according to a method reported earlier (Gottlieb, 1998). Kinematic and EMG data were compared with those obtained previously in a control group using identical experimental conditions (*n* = 8 right-handed healthy subjects, mean age ± *SD*: 34.8 ± 10.2 years; 3 women—values of this control group were used to compute z scores in the 3 experimental conditions) (see Manto et al., 2010).

### Statistical analysis

For both postural and action tremor, parameters in the post-sham and post-tCCDCS conditions were expressed as percentages of baseline values. For postural tremor, we computed the *z* scores as compared to patients' basal values. A *z* score between 1.959 *SD* above mean and 1.959 *SD* below mean corresponds to the 95% confidence interval (a *z* score between 2.576 *SD* above mean and 2.576 *SD* below mean corresponds to the 99% confidence interval). For fast goal-directed movements in the 3 conditions (basal, post-sham and post-tCCDCS), mean movement amplitudes and onset latencies of antagonist EMG activities were compared to data obtained in a control group reported earlier (see previous section) and expressed as *z* scores (Manto et al., 2010). To compare the relative entropy (sham/basal vs. tCCDCS/basal, bor both action tremor and postural tremor), we first applied the Shapiro-Wilk test to assess the normality. We subsequently used the Mann-Whitney rank sum test. Values were expressed as median ± *SD*. Statistical significance was set at 0.05.

## Results

### Clinical evaluation

In patient 1, the SARA rating score was:

*     -at baseline*: Gait (G) 3, Stance (St) 2, Sitting (Si) 4, Speech (Sp) 3, Finger chase (Fc): right 2 and left 2 (mean 2), Nose-finger test (Nf): right 1 and left 1 (mean 1), Fast alternating movements (Fa): right 1 and left 1 (mean 1), Heel-shin slide (Hs) right 2 and left 2 (mean 2). *Total*: 18;

*     -after sham*: G3, St2, Si2, Sp3, Fc: right 2 and left 2 (mean 2), Nf: right 1 and left 1 (mean 1), Fa: right 1 and left 1 (mean 1), Hs: right 2 and left 2 (mean 2). *Total*: 16;

*     -after tCCDCS*: G3, St2, Si2, Sp3, Fc: right 1 and left 2 (mean 1.5), Nf: right 1 and left 1 (mean 1), Fa: right 0 and left 1 (mean 0.5), Hs: right 2 and left 2 (mean 2). *Total*: 15.

     In patient 2, the SARA rating score was:

*     -at baseline*: G3, St2, Si1, Sp3, Fc: right 1 and left 1 (mean 1), Nf: right 1 and left 1 (mean 1), Fa: right 1 and left 1 (mean 1), Hs: right 1 and left 1 (mean 1). *Total*: 13;

*     -after sham*: G2, St2, Si2, Sp3, Fc: right 1 and left 1 (mean 1), Nf: right 1 and left 1 (mean 1), Fa: right 1 and left 1 (mean 1), Hs: right 1 and left 1 (mean 1). Total: 13;

*     -after tCCDCS*: G2, St1, Si1, Sp3, Fc: right 0 and left 1 (mean 0.5), Nf: right 1 and left 1 (mean 1), Fa: right 0 and left 0 (mean 0), Hs: right 1 and left 1 (mean 1). Total: 9.5.

### Upper limb tremor

#### Postural tremor

**Figure 2:**
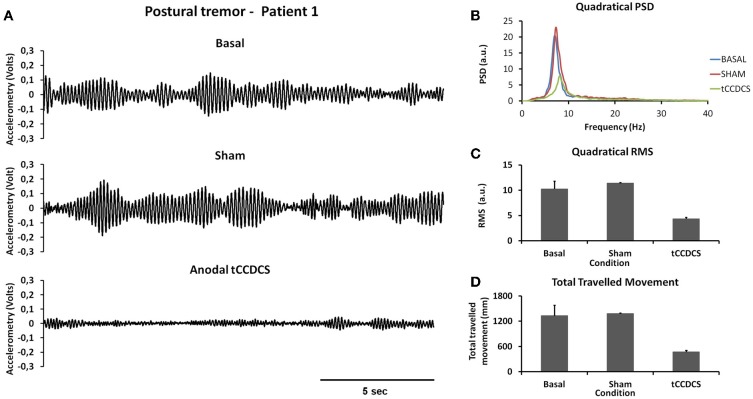
**Postural tremor in patient 1**. **(A)** Tremor recorded at the level of right index in the 3 experimental conditions (from top to bottom: baseline, post-sham and post-tCCDCS) for the gravity axis (dominant axis in this task). Calibration value: 1.55 mV = 100 mm/s^2^. **(B)** Mean quadratical power spectral density (PSD) of all epochs of postural tremor, showing a marked reduction of peak PSD following tCCDCS (blue line: baseline; red line: post-sham; green line: post-tCCDCS). Quadratical PSD is expressed in arbitrary units (a.u.). **(C)** Quadratical RMS (expressed in a.u.) of all epochs of postural tremor. (D) Total traveled movement (expressed in mm). Values in **(C)** and **(D)** are mean (±*SD*) of all epochs.

Figure 2A illustrates a representative recording of the postural tremor at the level of the index (accelerometer 1—gravity axis: axis with most intense oscillations in this task) in patient 1 in the 3 conditions (baseline, post-sham, post-tCCDCS). tCCDCS induced a major reduction of the amplitudes of the oscillations, unlike the sham procedure which did not modify tremor. PFr shifted from 7.1 Hz at baseline (post-sham: 7.2 Hz) to 8.3 Hz post-tCCDCS (*z* score: 2.97 as compared to baseline). The analysis of quadratical PSD of all epochs showed a drop of the peak PSD from 20.4 a.u. at baseline (post-sham: 23.0 a.u.) to 7.9 a.u. in the condition post-tCCDCS (38.63% of baseline values; see Figure 2B). The integral of the sub-band 4–20 Hz changed from 54.39 ± 7.26 a.u. (basal) to 60.52 ± 6.01 a.u. (post-sham; *z* score: 1.11 as compared to baseline) and to 29.23 ± 3.92 a.u. post-tCCDCS (*z* score: −3.46 as compared to baseline; drop to 53.74% of baseline values). CF decreased from 0.47 ± 0.04 to 0.39 ± 0.06 (post-sham; *z* score: −2 as compared to baseline), and to 0.28 ± 0.1 a.u. post-tCCDCS (z score: −3.71 as compared to baseline). Center frequency showed a trend to increase from 7.39 Hz (basal) to 8.6 Hz (post-tCCDCS) (*z* score: 2.36 as compared to baseline). Quadratical RMS changed from 10.36 ± 2.65 a.u. (basal) to 11.38 ± 0.27 a.u. (post-sham; *z* score: 0.42 as compared to baseline) and to 4.34 ± 0.48 a.u. post-tCCDCS (*z* score: −2.22 as compared to baseline) (see Figure 2C). Values for the total traveled movement are illustrated in Figure 2D. Very similar results were obtained for accelerometer 2 (data not shown).

**Figure 3:**
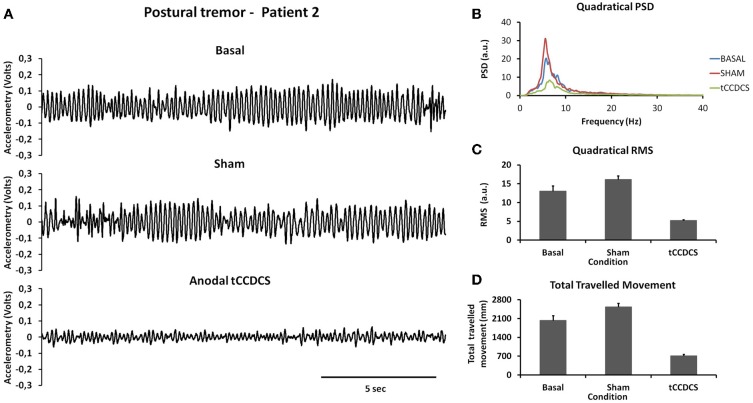
**Postural tremor in patient 2 (recordings at the right index; representative trace for the dominant axis in the gravity axis)**. **(A)** Tremor in the 3 experimental conditions. **(B)** Quadratical PSD. **(C)** Quadratical RMS. **(D)** Total traveled movement. See also legend of Figure 2 for details.

Similar observations were made for patient 2 (see Figure 3). Quadratical PSD showed a drop of the peak PSD from 20.3 a.u. at baseline (post-sham: 31.1) to 8.4 a.u. post-tCCDCS (41.42% of baseline values). The integral of the sub-band 4–20 Hz changed from 81.09 ± 11.97 a.u. (basal) to 96.65 ± 10.08 a.u. post-sham (*z* score: 1.29 as compared to baseline) and to 37.08 ± 4.94 a.u. post-tCCDCS (*z* score: −3.68 as compared to baseline). PFr, CF, and center frequency showed no significant changes in patient 2. Quadratical RMS changed from 13.0 ± 2.40 a.u. (basal) to 16.10 ± 1.75 a.u. post-sham (*z* score: 1.28 as compared to baseline) and to 5.19 ± 0.44 a.u. post-tCCDCS (*z* score: −3.25 as compared to baseline) (see Figure 3B).

#### Action tremor

**Figure 4:**
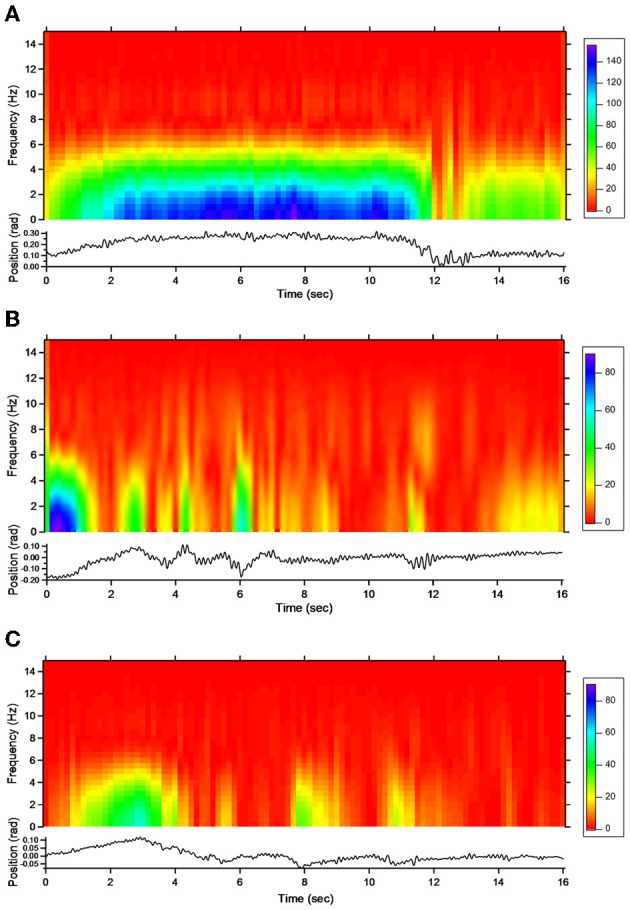
**Short-term Fourier transform (STFT) representation of action tremor in patient 1**. In each of the 3 panels (**A,B** and **C**), frequency (in Hz, upper) and position of the manipulandum (in rad, bottom) are illustrated as a function of time. **(A)** Baseline, **(B)** post-sham, **(C)** post-tCCDCS. Note that the color scale of the Y axis is larger in **(A)** as compared to **(B)** and **(C)**.

**Figure 5:**
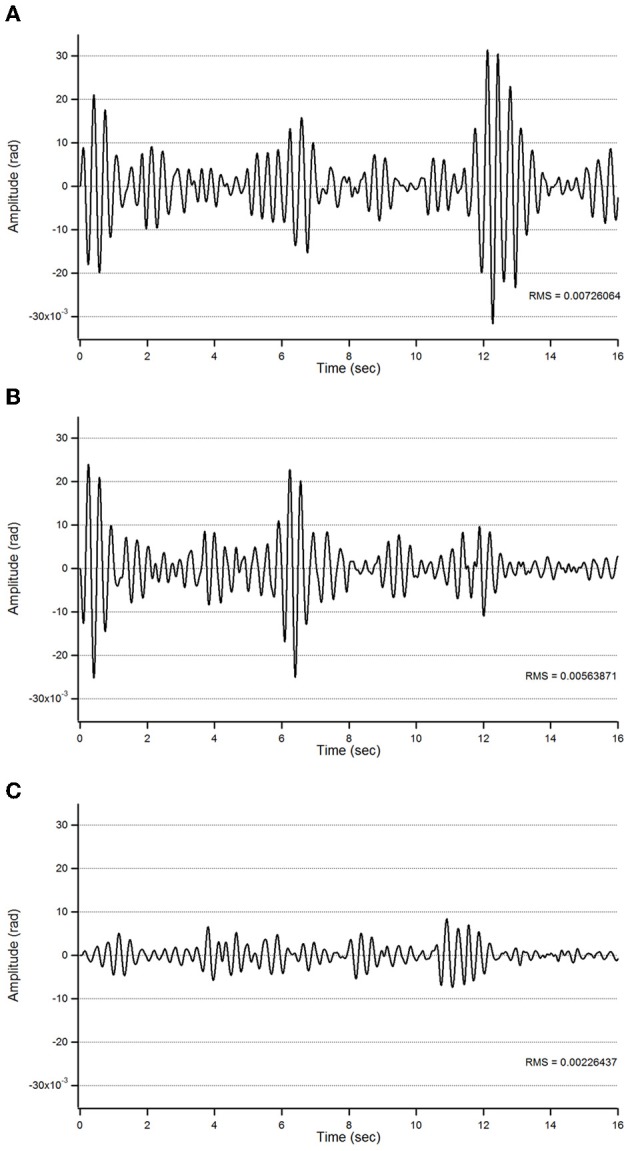
**Action tremor in the bandwidth 2.5–3.5 Hz in patient 1 in the 3 experimental conditions (A: basal; B: post-sham; C: post-tCCDCS) as a function of time (expressed in s)**.

For patient 1, the action tremor had a PFr of 7, 7.5, and 6.5 Hz, respectively, at baseline, post-sham and post-tCCDCS. The integral in the subband 4–20 Hz changed from 0.00769 a.u. (baseline) to 0.00695 a.u. (post-sham: 90.3% as compared to baseline) and to 0.00361 a.u. (post-tCCDCS: 46.9% as compared to baseline). At baseline, the STFT study showed that the oscillations were predominantly found below 5 Hz (Figure 4A). Although the sham procedure had a favorable effect on the magnitude of these low-frequency oscillations (Figure 4B), the effects were dramatic after tCCDCS (Figure 4C). These observations were confirmed by the analysis of tremor using a band-pass filter of 2.5–3.5 Hz (Figure 5). RMS decreased from 2.06 (basal; post-sham: 2.19) to 0.86 post-tCCDCS.

**Figure 6:**
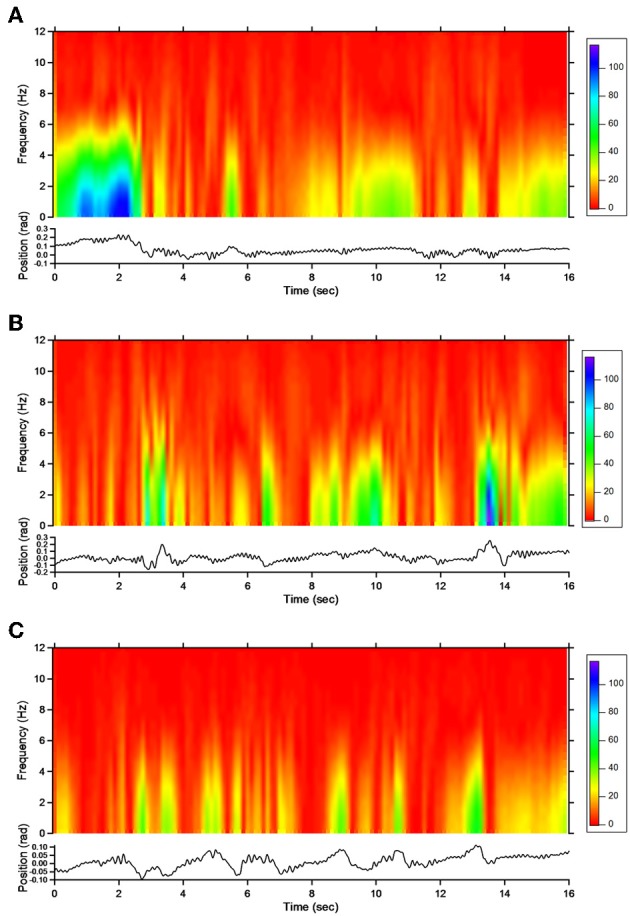
**STFT representation of action tremor in patient 2**. **(A)** Baseline, **(B)** post-sham, **(C)** post-tCCDCS. See also legend of Figure 4.

**Figure 7:**
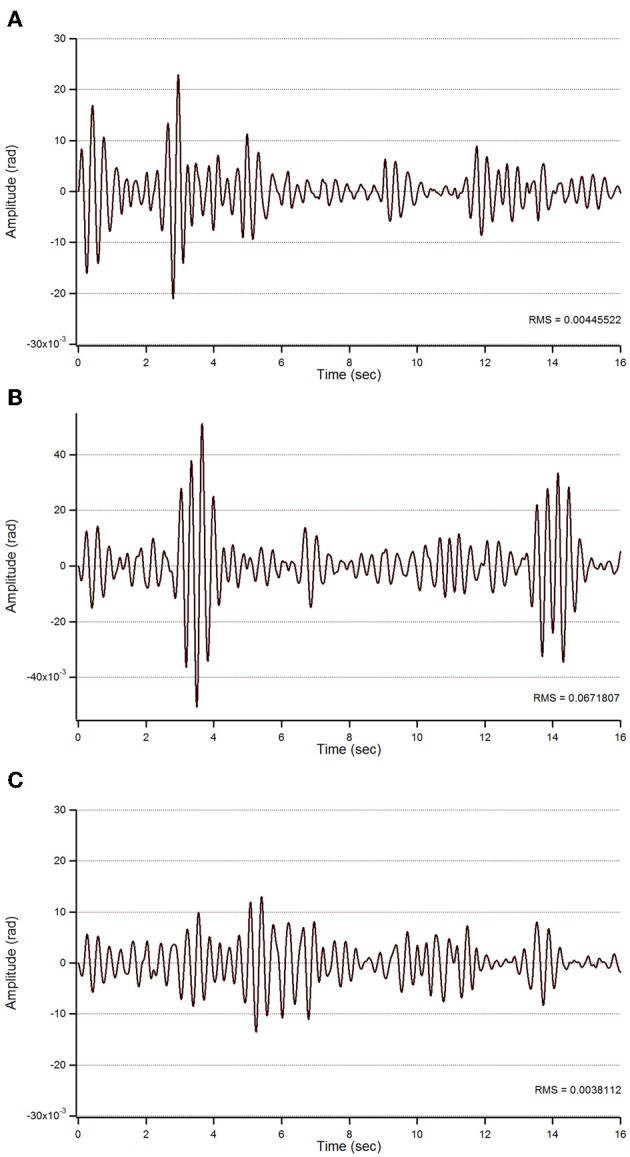
**Action tremor in the bandwidth 2.5–3.5 Hz in patient 2**. See also legend of Figure 5.

For patient 2, the action tremor had a PFr of 7, 7.05, and 1 Hz, respectively, at baseline, after sham and after tCCDCS. The integral in the subband 4–20 Hz changed from 0.01024 a.u. (baseline) to 0.01349 a.u. post-sham (131.7% as compared to baseline) and to 0.00638 a.u. post-tCCDCS (62.3% as compared to baseline). The STFT study also confirmed a favorable effect of tCCDCS on frequencies below 5 Hz, although at a lower extent as compared to patient 1 (Figure 6). These observations were confirmed by the analysis of tremor using a band-pass of 2.5–3.5 Hz (Figure 7). RMS decreased from 1.79 (basal; 2.84 post-sham) to 1.19 post-tCCDCS.

### Hypermetria

**Figure 8:**
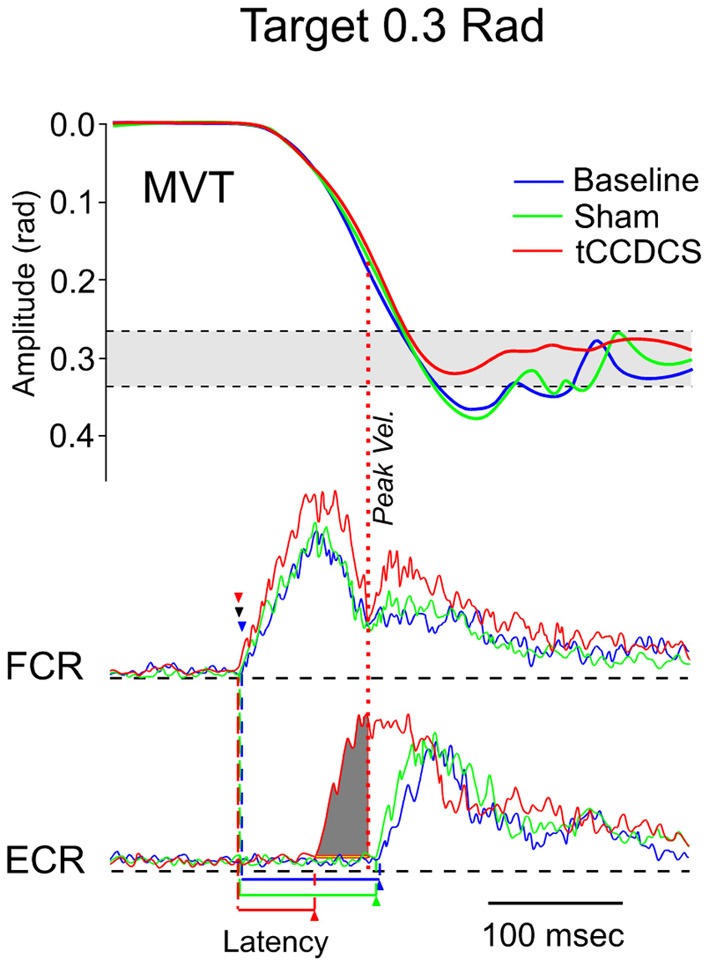
**Fast goal-directed wrist flexion movements in patient 1**. Target located at 0.3 rad from the start position. **Top part**: mean movement (MVT; averages of sets of 10 movements) in the 3 experimental conditions (blue line: basal condition, green line: sham, red line: post-tCCDCS); gray area corresponds to the mean ± 2.5 *SD* of position values obtained in a control group. **Middle** and **bottom part**: FCR and ECR correspond to averages (for the 10 movements) of full-wave rectified surface EMG activities of the flexor carpi radialis muscle (agonist muscle) and extensor carpi radialis muscle (antagonist muscle), respectively (blue lines: basal condition, green lines: sham, red lines: post-tCCDCS). Arrowheads indicate the onset of EMG activities (blue: baseline; green: post-sham; red: post-tCCDCS). Note that the burst of antagonist EMG activity starts earlier after tCCDCS, demonstrating an effect of tCCDCS on the timing of antagonist EMG activity. The dark gray area below the ECR trace corresponds to the portion of EMG activity occurring *before* the peak velocity (illustrated by a red dotted line, whereas the peak velocity for the baseline and post-sham condition occurred *before* the onset latency of the antagonist EMG activity), and thus having an efficient braking action to avoid the overshoot of the target.

**Figure 9:**
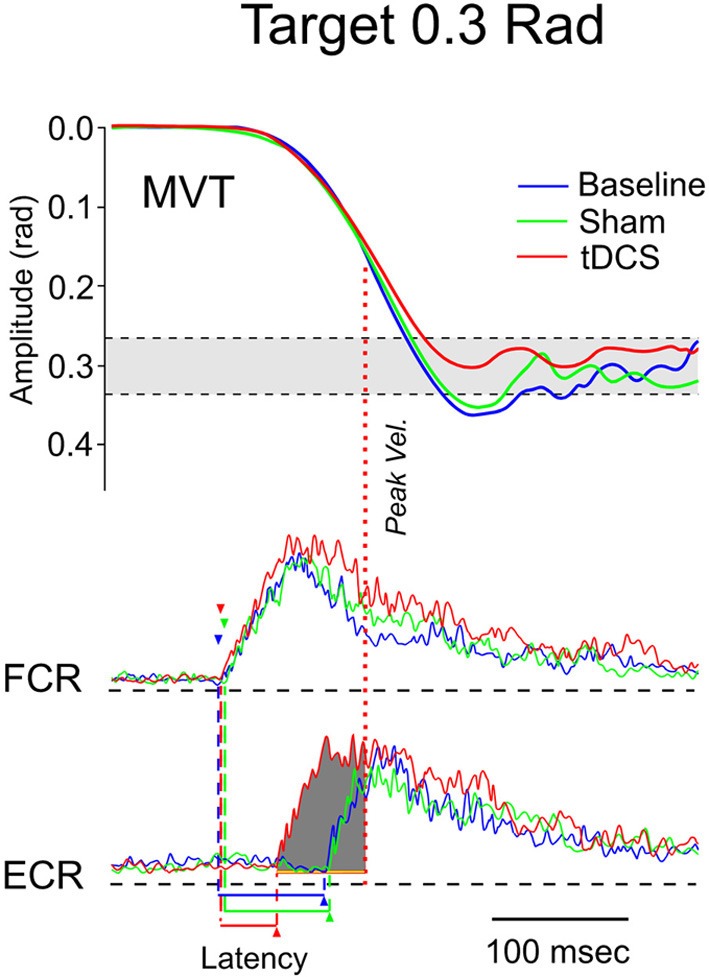
**Fast goal-directed wrist flexion movements in patient 2 (target: 0.3 rad from the start position)**. **Top part:** mean movement (MVT) in the 3 experimental conditions (blue line: basal condition, green line: sham, red line: post-tCCDCS). **Middle** and **bottom part:** FCR and ECR correspond to surface EMG activities of the flexor carpi radialis muscle and extensor carpi radialis muscle, respectively (blue lines: basal condition, green lines: sham, red lines: post-tCCDCS). Note the reduction of the onset latency of the antagonist EMG activity following tCCDCS. See also legend of Figure 8 for details.

In both patients, fast goal-directed movements were markedly hypermetric at baseline and following application of the sham procedure (see Table 1). In both patients, hypermetria was associated with a delayed onset latency of the antagonist EMG activity. After application of tCCDCS, a dramatic effect on hypermetria was observed, with a clear reduction of the onset latency of the antagonist EMG activity. This is illustrated in Figure 8 in patient 1 for the aimed target of 0.3 rad. Figure 9 illustrates similar observations in patient 2 (aimed target of 0.3 rad). Considering the 3 aimed amplitudes and the 2 patients, the *z* scores for the values of the onset latency of the antagonist activity were (mean ± *SD*): 7.12 ± 0.69 at baseline, 6.79 ± 0.71 post-sham (95.5% as compared to baseline), and 1.28 ± 1.27 post-tCCDCS (18.8% as compared to baseline).

**Table 1:** **Kinematic and EMG parameters associated with fast goal-directed movements in the 2 patients**.

**Kinematic and EMG parameter**	**Condition**	**Target: 0.2 rad**		**Target: 0.3 rad**		**Target: 0.4 rad**	
		Patient 1 (*z* score)^*,#^	Patient 2 (*z* score)^*,#^	Patient 1 (*z* score)^*,#^	Patient 2 (*z* score)^*,#^	Patient 1 (*z* score)^*,#^	Patient 2 (*z* score)^*,#^
Mean movement Amplitude (rad)^a^	Basal	0.2948 ± 0.0512	0.2601 ± 0.035	0.3679 ± 0.0254	0.3633 ± 0.0241	0.4720 ± 0.018	0.4678 ± 0.0268
		(7.53)^#^	(4.79)^#^	(4.76)^#^	(4.43)^#^	(5.43)^#^	(5.12)^#^
	Sham	0.2985 ± 0.0480	0.2519 ± 0.0292	0.3788 ± 0.0288	0.3527 ± 0.0231	0.4630 ± 0.025	0.4557 ± 0.0233
		(7.82)^#^	(4.10)^#^	(5.53)^#^	(3.68)^#^	(4.76)^#^	(4.21)^#^
	tCCDCS	0.2191 ± 0.0193	0.2108 ± 0.021	0.3222 ± 0.0156	0.3023 ± 0.0185	0.4118 ± 0.016	0.4118 ± 0.0162
		(1.48)	(0.81)	(1.53)	(0.12)	(0.94)	(0.94)
Onset latency of antagonist EMG activity (ms)^b^	Basal	98	87	105	78	108	74
		(7.75)^#^	(6.38)^#^	(7.22)^#^	(4.22)^#^	(5.73)^#^	(2.64)^#^
	Sham	95	84	103	77	101	72
		(7.38)^#^	(6.0)^#^	(7.0)^#^	(4.11)^#^	(5.09)^#^	(2.45)
	tCCDCS	63	43	59	41	57	46
		(3.38)^#^	(0.88)	(2.11)	(0.11)	(1.09)	(0.09)

* *As compared to values obtained in a control group (n = 8 subjects; see also Methods)*.

# Values are outside the 99% confidence interval of control values.

a Control values: target at 0.2 rad: 0.2006 ± 0.01251 rad; target at 0.3 rad: 0.3006 ± 0.01451; target at 0.4 rad: 0.3992± 0.01341.

b Control values: target at 0.2 rad: 36 ± 8 ms; target at 0.3 rad: 40 ± 9 ms; target at 0.4 rad: 45 ± 11 ms.

### Relative entropy

**Figure 10:**
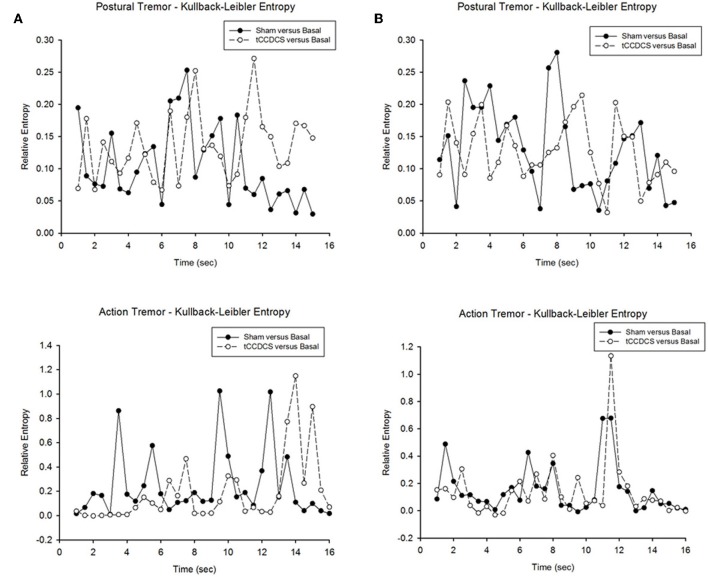
**Relative entropy in patient 1 (A) and patient 2 (B)**. Upper panels correspond to the relative entropy for postural tremor in the condition sham vs. basal (filled circles) and tCCDCS vs. basal (open circles—dotted lines). Bottom panels correspond to the relative entropy for action tremor in the condition sham vs. basal (filled circles) and tCCDCS vs. basal (open circles—dotted lines).

In both patients, we found a strong overlap in terms of relative entropy of tremor (Figure 10). However, a statistically significant increase in relative entropy was found for postural tremor in patient 1 (median ± *SD*: 0.085 ± 0.061 vs. 0.131 ± 0.052; *p* = 0.031). Still, no difference was found for action tremor: median values (±*SD*) were 0.153 (±0.279) and 0.066 (±0.28) in sham/basal vs. tCCDCS/basal, respectively (*p* = 0.059). For patient 2, relative entropies were similar for postural tremor (0.129 ± 0.069 vs. 0.125 ± 0.048; *p* = 0.988) and for action tremor (0.086 ± 0.183 vs. 0.077 ± 0.213; *p* = 0.593).

## Discussion

Our observation highlights a novel way to modulate cerebellar motor functions in a neurological disorder manifesting with a predominant cerebellar syndrome. This is the first demonstration that tCCDCS could be considered as a symptomatic therapeutic strategy of upper limb deficits in a disabling cerebellar ataxia. In our 2 patients, tCCDCS improved clinically the voluntary movements of the upper limbs, without affecting oculomotor disturbances, dysarthria and ataxia of stance/gait as confirmed by the ataxia rating scale. Interestingly, both patients reported a feeling of motor improvement after tCCDCS.

The excitability of the cerebellar circuitry is impaired in numerous cerebellar disorders, as a result of the degenerative process occurring in the cerebellum and its afferences (Wessel et al., 1996). In SCAs, the degenerative process even extends beyond cerebellar circuits. The sole application of tDCS at the level of the cerebellum in ataxic patients has not shown functional effects so far. Although anodal tDCS of the cerebellum reduces the magnitudes of long-latency stretch reflexes (LLSR) in sporadic cerebellar atrophy or in dominant ataxias (Grimaldi and Manto, 2013), it does not improve coordination in upper limbs or postural ataxia. The enhanced LLSR in ataxic patients are explained by a disinhibition of cerebellar nuclei as a result of a damage of the cerebellar cortex (Diener et al., 1984). Anodal tDCS of the cerebellum reinforces the inhibitory tone exerted by the cerebellar cortex over cerebellar nuclei, but these effects alone seem insufficient to improve cerebellar ataxia. Effects on brainstem nuclei are unlikely since cerebellar tDCS does not modify the excitability of vestibular or trigeminal nuclei (Galea et al., 2011; Jayaram et al., 2012). Modeling studies show that the diffusion of electric field outside the cerebellum is small (Parazzini et al., 2013). We have suggested that anodal tDCS of the cerebellum does not improve functional tests because these tasks depend on the integrity of diffuse cerebello-cerebral networks whose activity is not modified substantially by anodal tDCS applied over the cerebellum in a spatially-restricted manner, as used here (Grimaldi and Manto, 2013). The combination of tDCS of the cerebellum with tDCS of the motor/premotor cortex is thus emerging as a possible novel approach in the field of cerebellar neuromodulation.

The delayed onset latency of the antagonist EMG activity associated with fast goal-directed movements is a major finding in patients with a cerebellar disorder (Hore et al., 1991; Manto et al., 1994). The parameter is robust for the follow-up of cerebellar patients and is correlated with the clinical deficits (Manto et al., 1995). The variability of the onset latency of antagonist EMG activity is less than 7% when fast goal-directed movements are performed at the same peak velocities. It is assumed that the delayed onset of the braking action of the antagonist activity results in the hypermetria, a cardinal feature of genuine cerebellar diseases. So far, attempts to improve this key-parameter of the timing of antagonist commands to voluntary muscles with the use of drugs have failed. Cooling the dentate nucleus in monkeys induces hypermetria (Flament and Hore, 1986). The overshoot is associated with a delay in the generation of the antagonist muscle activity *and* abnormal neuronal discharges in the contralateral motor cortex (Hore and Flament, 1988). This distorted timing of muscle discharges is considered as a signature of a cerebellar lesion involving the cerebello-thalamo-cortical pathway. We suggest that tCCDCS restores this property of the cerebello-thalamo-cortical pathway. The internal representation of temporal information would be modified by tCCDCS (Ivry and Spencer, 2004). Consequently, predictions of body states would be improved (Molinari et al., 2009).

At a first glance, a strong overlap was found between sham and tCCDCS in terms of entropy. Still, the relative entropy was increased in one patient following tCCDCS in the postural condition. However, this is an avenue for future studies. Indeed, subsequent works are required to assess whether tCCDCS really impacts on the randomness of neurological tremor. The study of relative entropy parameter has not been exploited so far in the field of ataxic disorders, despite (a) the critical roles of the cerebellum in the programming of timing and sequencing of motor operations, and (b) the major contributions of cerebellar circuits in the so-called Guillain-Mollaret triangle implicated in tremor genesis (Vidailhet et al., 1998; Molinari et al., 2009).

A severe postural tremor highly responsive to subthalamic-thalamic deep brain stimulation (DBS) has been reported in a patient with SCA2 (Freund et al., 2007). However, DBS is usually not considered as a primary option in the management of tremor in SCA2, especially because the disease is progressive and the surgical procedure carries several risks. Nevertheless, the previously reported excellent response to DBS also underlines that cerebellar efferences are critical to cause limb oscillations.

Although our results are very encouraging, there are limitations to our study: our number of patients is small, we did not address the pathogenesis of other cerebellar deficits such as gait deficits and we did not investigate specifically the dynamics of the duration of the after-effects. Future studies should be performed in larger groups of ataxic patients not only to confirm our findings in the numerous forms of cerebellar ataxias, but also to identify the categories of ataxic patients which can be considered as potential responders. tDCS is non-invasive and more extensive electrodes covering the skull over the posterior fossa can be envisioned. These electrodes might have the advantage of modulating the entire cerebellar cortex and could be characterized by a spatial map allowing a dynamic application of currents, with the intensity of currents being adapted to the region of the cerebellum targeted by tDCS as well as to the task. Future studies are required to validate this “*whole cerebellum DCS*” strategy in cerebellar ataxias. The mechanisms and duration of the after-effects deserve attention (Márquez-Ruiz et al., 2012). At the cerebral cortex level of behaving rabbits, tDCS modifies thalamo-cortical synapses by acting principally at presynaptic sites and cathodal tDCS appears more effective than the anodal tDCS in terms of duration of the after-effects (Márquez-Ruiz et al., 2012). In healthy subjects, cathodal tDCS of the cerebellum lasts up to 30 min after the cessation of stimulation in a paradigm of cerebellum-brain inhibition (CBI) evaluated with transcranial magnetic stimulation (TMS) (Galea et al., 2009). Other studies point out that anodal tDCS induces significant and long-lasting effects (more than 24 h) in models of pain related to peripheral inflammation in rats (Laste et al., 2012). Both immediate effects and after-effects are influenced by stimulus parameters (pulse shape, intensity, duration, and frequency of stimuli) and electrodes (location, size, and orientation). The non-linear relationships between stimuli and physiological responses should be taken into account (Paulus et al., 2013). The region of the brain which is stimulated is another factor. For instance, the cerebellum has a distinct electrical conductivity and permittivity—a measure of how an electric field influences a dielectric medium—as compared to the brainstem or cerebrum (Nightingale et al., 1983). The question of the use of repeated administration of tDCS in chronic disorders of the brain remains open at this stage. In particular, it is difficult to anticipate whether tDCS will become a technique applicable repeatedly to patients with chronic cerebellar atrophy. Safety studies as well as efficacy studies are needed. In terms of safety, several studies addressing the effects of tDCS on cerebellar function have typically used a current intensity of 2 mA, which is often the intensity associated with prickling sensations at the skin or a mild discomfort (Tomlinson et al., 2013). Recent works in chronic aphasic patients underline that tDCS potentiates the recovery of clinical deficits, but long-term studies with repeated sessions of stimulation are still missing (Marangolo et al., 2014).

Cerebellum is part of a distributed cognitive network and its neuromodulation influences the contribution of cerebellar circuits, especially for linguistic processes, working memory tasks and processing of emotions (Ferrucci et al., 2008; Argyropoulos et al., 2011; Pope and Miall, 2012). This extends strongly the potential applications. The impacts of using non-invasive neurostimulation such as tDCS techniques over the cerebellum in cognitive and psychiatric rehabilitation strategies, as well as the long-term neural consequences need to be clarified in rigorous large scale trials (Grimaldi et al., 2014).

## Author contributions

Overall study design and protocol development: Giuliana Grimaldi, Nordeyn Oulad Ben Taib, Mario Manto. Data analysis: Giuliana Grimaldi, Mario Manto, Florian Bodranghien. Writing of manuscript: Giuliana Grimaldi, Nordeyn Oulad Ben Taib, Mario Manto, Florian Bodranghien. Final version reviewed and approved by all the authors.

### Conflict of interest statement

The authors declare that the research was conducted in the absence of any commercial or financial relationships that could be construed as a potential conflict of interest.
